# Heat Shock Signaling in Land Plants: From Plasma Membrane Sensing to the Transcription of Small Heat Shock Proteins

**DOI:** 10.3389/fpls.2021.710801

**Published:** 2021-08-09

**Authors:** Baptiste Bourgine, Anthony Guihur

**Affiliations:** Department of Plant Molecular Biology, Faculty of Biology and Medicine, University of Lausanne, Lausanne, Switzerland

**Keywords:** heat shock transcription factor, heat shock response, acquired thermotolerance, cyclic nucleotide-gated channels, calmodulins, small heat-shock proteins, heat stress, global warming

## Abstract

Heat stress events are major factors limiting crop productivity. During summer days, land plants must anticipate in a timely manner upcoming mild and severe temperature. They respond by accumulating protective heat-shock proteins (HSPs), conferring acquired thermotolerance. All organisms synthetize HSPs; many of which are members of the conserved chaperones families. This review describes recent advances in plant temperature sensing, signaling, and response. We highlight the pathway from heat perception by the plasma membrane through calcium channels, such as cyclic nucleotide-gated channels, to the activation of the heat-shock transcription factors (HSFs). An unclear cellular signal activates HSFs, which act as essential regulators. In particular, the HSFA subfamily can bind heat shock elements in HSP promoters and could mediate the dissociation of bound histones, leading to HSPs transcription. Although plants can modulate their transcriptome, proteome, and metabolome to protect the cellular machinery, HSP chaperones prevent, use, and revert the formation of misfolded proteins, thereby avoiding heat-induced cell death. Remarkably, the HSP20 family is mostly tightly repressed at low temperature, suggesting that a costly mechanism can become detrimental under unnecessary conditions. Here, the role of HSP20s in response to HS and their possible deleterious expression at non-HS temperatures is discussed.

## Introduction

During summer days, mild or severe heat stress (HS) typically occurs at midday and lasts until late afternoon in terrestrial systems (Dong et al., 2017). To survive, a plant must sense, early in the morning, a minor temperature increment to establish a suitable genetic program. The heat shock response (HSR) contains molecular defenses, including heat shock proteins (HSPs), that must accumulate rapidly under rising temperatures to minimize foreseeable damage (Song et al., 2012; Serrano et al., 2019). Acquired thermotolerance (AT) refers to the plant adaptive capacity to survive noxious HS when exposed to sublethal temperatures, requiring the accumulation of HSPs. Under HS, both transcriptome and proteome-based studies have indicated regulatory responses of HSPs (Qin et al., 2008; Finka et al., 2011; Mangelsen et al., 2011; Xin et al., 2016; Guihur et al., 2020; Zhao et al., 2021). A conserved subfamily called “heat-induced molecular chaperones” contains the HSP100s, HSP90s, HSP70s, HSP60s, HSP40s, and HSP20s (Al-Whaibi, 2011; Jee, 2016). They are 20 times more likely to be heat-induced compared to non-chaperone proteins (Wang et al., 2004; Finka et al., 2011, 2015; Guihur et al., 2020). HSP chaperones prevent and repair protein misfolding and aggregation, reducing cell damage (Ben-Zvi and Goloubinoff, 2001; Wang et al., 2004; Zeng et al., 2004; Liberek et al., 2008; Mogk and Bukau, 2017). In particular, HSP20s are the most heat-responsive in plants due to their dramatic induction (Vierling, 2003; Guihur et al., 2020). They also prevent the aggregation of heat-labile proteins and could stabilize lipids at the plasma membrane (Haslbeck and Vierling, 2015). At low temperature, HSP20s are tightly repressed, suggesting that their inappropriate expression could be deleterious for plants (Sun et al., 2016). Moreover, HS generates stress granules that contain molecular chaperones, such as HSP20s, HSP101, untranslated mRNAs, elongation initiation factors, RNA-binding proteins and transcription factors (McLoughlin et al., 2016, 2019; Chantarachot and Bailey-Serres, 2018; Kosmacz et al., 2019). Theses cytoplasmic and chloroplastic bodies seem to have an important role in protein translation during and after HS (Merret et al., 2017; Chodasiewicz et al., 2020).

In most eukaryotes, including land plants, HSP accumulation depends on a signal that arises at the plasma membrane and results in the activation of heat shock transcription factor (HSF) families (Nover et al., 2001; Mishra et al., 2002; Hayashida et al., 2011; Liu et al., 2011; Scharf et al., 2012; Fragkostefanakis et al., 2015; Kijima et al., 2018). Plant cells can sense a wide temperature range through changes in the plasma membrane fluidity. Calcium channels, such as cyclic nucleotide-gated channels (CNGCs), can mediate calcium entry during HS as shown in *Arabidopsis thaliana* and *Physcomitrium patens* (Gong et al., 1998; Saidi et al., 2009; Finka et al., 2012; Gao et al., 2012; Tunc-Ozdemir et al., 2013). Yet, a fraction of HSFA1 is associated with the HSP70-HSP90 complex, and a large inactivated fraction might remain unbound under non-stressful conditions (Kyle Hadden et al., 2006; Westerheide et al., 2006; Saidi et al., 2009; Hahn et al., 2011). Following the activation of CNGCs, and a still unclear signaling pathway, HSFA1 is translocated into the nucleus and can bind specific DNA motifs present in the promoter of HSP genes, called “heat shock element” (HSE) (Santoro et al., 1998; Liu and Charng, 2012). HSFA1 could also trigger regulatory responses, including DNA methylation, histone modification, and chromatin remodeling (Zhao et al., 2021). In particular, bound histones to HSP genes, such as H2A.Z, must be evicted to allow for RNA polymerase II docking for the transcription of HSP (Franklin, 2010; Kumar and Wigge, 2010; Probst and Mittelsten Scheid, 2015). Yet, the mechanisms of heat sensing, particularly the components between the CNGCs sensors and the activation of the main regulator HSFA1 are not elucidated (Larkindale et al., 2005; Mittler et al., 2012). This review addresses the heat perception and signaling pathway in land plants, with a particular emphasis on the activation of HSFA1 at the plasma membrane, leading to the accumulation of HSP chaperones. In addition, the role of HSP20s at non-HS temperatures and their putative effect in plant cells are discussed. Understanding these critical processes would facilitate the production and selection of thermotolerant cultivars to face global warming.

## Heat Sensing and Signaling in Land Plants

### Calcium Entry Across the Plasma Membrane Triggers the Heat Shock Signaling

**FIGURE 1:**
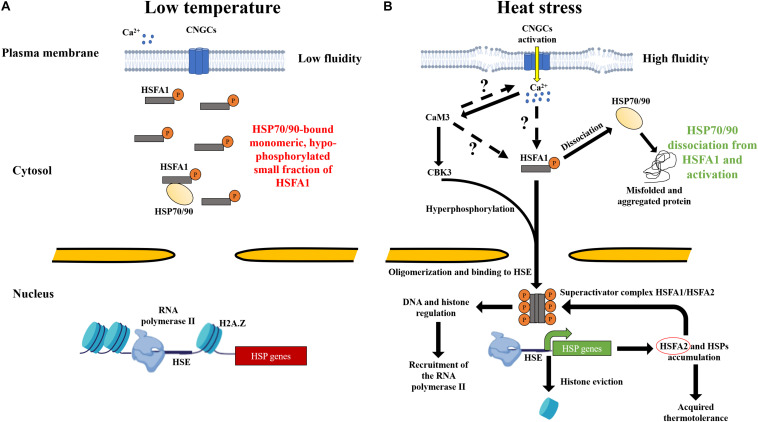
Heat perception by the plasma membrane and mechanisms leading to the onset of acquired thermotolerance in land plants. **(A)** At low temperature, the rigidity of the plasma membrane prevents the activation of hypophosphorylated key regulator HSFA1, while a small fraction might still bind the HSP70-HSP90 complexes in the cytosol. Histones, such as H2A.Z, associate and condense the DNA and prevent the RNA polymerase from accessing HSP genes for active transcription. **(B)** The increase in temperature increases the fluidity of the plasma membrane, resulting in the activation of CNGCs heat sensors. Transient periplasmic Ca^2+^ entry into the cytosol trigger is an unknown signaling cascade that activates HSFA1. Excessive temperatures are expected to denature heat-labile proteins in the cytosol. Misfolded and aggregated proteins are assumed to recruit the HSP90 and HSP70 being in part bound to HSFA1. The dissociation of HSP70-90 is a key step that leads hyperphosphorylation of HSFA1 by CBK3 and leads to its translocation into the nucleus to bind HSEs. HSFA1 might mediate a signal to the chromosome remodeling machinery to remove bound histones from HSP genes. HSFA1 can also activate histone and DNA modifications required for the regulatory responses of HSPs. RNA polymerase II is then recruited for the transcription of HSFA2 (red circle), forming a superactivator complex with HSFA1 and leading to accumulation of HSPs, ultimately conferring acquired thermotolerance in land plants.

Plant cells developed an efficient mechanism for sensing the increase in temperature, as well as a signaling cascade for a rapid adaptive response. The nucleus, endoplasmic reticulum, cytosol, mitochondria, and chloroplast may also contain heat sensors (Bussell et al., 2010; Franklin, 2010; Schwarzländer and Finkemeier, 2012; Hentze et al., 2016; Sun and Guo, 2016; Chang et al., 2017; Lin K. F. et al., 2018). However, various observations have indicated that the primary heat sensing might occur at the plasma membrane. For instance, electrophysiology measurements in *P. patens* protoplasts, expressing the calcium-sensitive fluorescent protein aequorin reporter, have demonstrated a saturated accumulation of cytosolic Ca^2+^ within the first 10 min at 38°C (Saidi et al., 2009). Artificially preventing the entry of periplasmic Ca^2+^ in *A. thaliana* and *P. patens* protoplast showed a lack of HSP expression. A defective HSR has also been described in the presence of ionomycin and thapsigargin, which are ionophores known to release Ca^2+^ from internal stores (Saidi et al., 2009; Finka et al., 2012). Similar observations were reported for tobacco, maize, and rice (Gong et al., 1998; Li et al., 2004; Wu and Jinn, 2010; Wu et al., 2012). Yet, this phenotype has not been observed in *Chlamydomonas reinhardtii*, suggesting another mechanism of heat perception in green algae (Schmollinger et al., 2013). Thus, the HSR seems to depend on Ca^2+^ entry across the plasma membrane in land plants (Figure 1; Demidchik et al., 2018).

### Embedded Cyclic Nucleotide-Gated Channels Act as Plasma Membrane Thermosensors

In both plants and animals, plasma membrane contains CNGCs, which are tetrameric cation channels and contain six transmembrane domains. They modulate Ca^2+^ entry from the apoplast and other ions, such as Mg^2+^, K^+^, Na^+^, or Pb^+^ (Véry and Sentenac, 2002). CNGCs can be assembled as homotetrameric or heterotetrameric complexes, allowing for the formation of a large array of sensors capable of responding to different intensities of environmental cues (Clough et al., 2000; Tan et al., 2020). The cytosolic C-terminus harbors a cyclic nucleotide-binding domain (CNBD) and a calmodulin-binding domain (CaMBD), in which a calmodulin (CaM) binding isoleucine-glutamine (IQ) motif is embedded (Kaplan et al., 2007; Jarratt-Barnham et al., 2021). *A. thaliana* AtCNGC2 and four acts as a heat sensor since its lack of expression leads to a hyper thermosensitive phenotype, resulting in a higher accumulation of HSPs at lower temperature. Similar results were observed in the orthologs CNGCb and CNGCd of *P. patens* (A Finka et al., 2012; Finka and Goloubinoff, 2014). In addition, the lack of AtCNGC6 in *A. thaliana* leads to a fewer transcript levels of HSP18.2, HSP25.3, and HSP70 compared to control plants at 37°C, ultimately impacting the HSR (Gao et al., 2012). In rice, reduced thermotolerance was observed in *Oscngc14* and *Oscngc16* mutants, resulting in a higher accumulation of hydrogen peroxide, leading to the cell death (Cui et al., 2020). These results strongly suggest that CNGCs act as thermosensors in land plants. Other calcium channel families have been also implicated in heat response. For example, the lack of synaptotagmin A activity led to decreased HSPs synthesis in *A. thaliana* at 45°C (Yan et al., 2017). ANNEXIN calcium channels can modulate cytosolic calcium signature under oxidative and heat stresses (Liao et al., 2017). Glutamate receptor-like channels have been suggested to participate in Ca^2+^ signaling since exogenous glutamate improves basal thermotolerance in maize (Li et al., 2019). Therefore, the heat sensing by the plasma membrane of plants contains calcium channels, including CNGCs, that can respond to incremental temperatures, mediating Ca^2+^ entry and triggering the signaling pathway for the accumulation of HSPs.

### Calmodulins Response to Heat

The signaling molecules located in the cytosol and required to activate HSFA1s are not yet uncovered (Figure 1). Yet, both CNBD and CaMBD present on the cytosolic part of CNGCs suggest that cyclic nucleotide monophosphate and CaMs can mediate the heat signaling (Gao et al., 2012). CaMs are made of calcium-binding loops, called “E” and “F” that can each bind two Ca^2+^ ions and can respond to biotic and abiotic stress in plants (Rhoads and Friedberg, 1997; Mccormack et al., 2005; Fischer et al., 2013; Virdi et al., 2015). *A. thaliana* contains nine CaMs; among them are the first seven, which are highly conserved. In addition, 50 members of calmodulin-like proteins (CMLs) have been described as important players in stress perception and plant development (Aldon and Galaud, 2006; Vadassery et al., 2012). Interestingly, AtCaM2, AtCaM4, AtCaM6, AtCaM7, and AtCML8 were found to bind the C-terminal of several CNGC families (Fischer et al., 2017). At 37°C, AtCNGC6 was negatively regulated by AtCaM2/3/5 and AtCaM7, which interact with the IQ motif of AtCNGC6 and impact Ca^2+^ entry (Niu et al., 2020). A potentially important role in the heat signaling has been demonstrated for *AtCaM3* where the knockout mutant has shown reduced levels of HSP18.2 and HSP25.3 transcripts at 37°C, negatively impacting basal thermotolerance. The overexpression of AtCaM3 leads to a significant increase in the HSPs level and improves the resistance against noxious temperatures (Zhang et al., 2009). AtCaM3 has been also suggested to activate several components of the heat shock signaling pathway, such as mitogen-activated protein kinase 6 and calmodulin-binding protein kinase 3 (CBK3) (Figure 1; Liu et al., 2005; Yan et al., 2017). Moreover, AtCBK3 promotes HSFA1 activation by phosphorylation. Under HS, the lack of AtCKB3 dramatically reduced HSP18.2 and HSP25.3 levels, resulting in defective basal thermotolerance, whereas the *Atcbk3* overexpression line rescued the hypersensitivity phenotype (Liu et al., 2008; Yip Delormel and Boudsocq, 2019). In other plant species, several CaMs have been described to mediate the heat signal. In rice, OsCaM1-1 was shown to positively regulate Ca^2+^ signals, resulting in HSP accumulation (Wu and Jinn, 2012; Wu et al., 2012). In wheat, CaM1-2 has been found to act upstream of HSP26 and HSP70 at 37°C (Liu et al., 2003). Therefore, CaMs have been mentioned to play a critical role in the heat signaling of land plants and responding to other environmental stimuli (Virdi et al., 2015). Yet, other components of the heat signaling pathway between CNGC sensors and HSFA1s remain to be identified (Figure 1).

### Heat Shock Transcription Factor A1 Acts as a Key Regulator of the Heat Shock Signaling Pathway

HSFs are essential regulators of the heat signaling pathway in many organisms (Gallo et al., 1993; Mishra et al., 2002; Nicholls et al., 2009; Anckar and Sistonen, 2011). In contrast to vertebrates, which contain fewer members (six for humans), plant HSF families have more members that reflect their strategy for a sessile adaptation in changing environment (von Koskull-Doring et al., 2007; Huang et al., 2016; Gomez-Pastor et al., 2018). For example, *A. thaliana* contains 21 HSFs, 24 for tomato, 52 for soybean, or 56 for wheat (Scharf et al., 2012; Xue et al., 2014; Fragkostefanakis et al., 2015). Plant HSFs are classified into three classes: HSFA, B, and C. All HSFs have a DNA binding, oligomerization, and nuclear localization domains. Yet, HSFAs differ in the presence of an activator region at the C-terminal, which binds HSEs, whereas HSFBs contain an inhibitor region. Under HS, several subfamilies of HSFA are required for the accumulation of HSPs. HSFB contains subfamilies, which can act as coactivators of HSPs transcription and, also, as antagonist repressors, competing for HSFAs at the end of HS (Czarnecka-Verner et al., 1997; Santoro et al., 1998; Mishra et al., 2002; Mitsuda and Ohme-Takagi, 2009; Ikeda et al., 2011; Scharf et al., 2012; Liu and Charng, 2013; Fragkostefanakis et al., 2015; Guo et al., 2016). In *A. thaliana*, the *hsfA1a* mutant was shown to be ineffective in accumulating several HSP transcripts at 37°C, resulting in the absence of AT (Liu et al., 2011). Similar observations were previously made in tomato where HSFA1 has been shown to be a master regulator for AT (Mishra et al., 2002). Studies in several monocotyledonous species, such as wheat and rice, have also demonstrated the important role of HSFA in mediating the heat signal (Yokotani et al., 2008; Zhang et al., 2013; Guo et al., 2016). Furthermore, the role of HSFA1 is not only limited to the transcription of HSPs, but it also activates several transcription factors, such as HSFA2, HSFA3, HSFA7a, multiprotein bridging factor 1C, and dehydration-responsive element-binding protein 2A, which are required for HSP synthesis and thermotolerance in *A. thaliana* (Suzuki et al., 2011; Yoshida et al., 2011; Liu and Charng, 2013; Ohama et al., 2017). When accumulated, HSFA2 can form a heterodimer with HSFA1 and thereby forming a superactivator complex for sustaining HSPs expression under HS (Chan-Schaminet et al., 2009). Thus, the family of HSFAs has been identified as a major regulator required for the onset of AT in land plants (Mishra et al., 2002; Hahn et al., 2011; Yoshida et al., 2011).

At low temperature, inactive cytosolic HSFA1s are hypophosphorylated and bound to the complex HSP70-HSP90 (Figure 1; Hahn et al., 2011; Morimoto, 2012). The traditional model suggests that, upon HS, HSP70-HSP90 complex is hijacked by the increased cytosolic levels of unfolded or misfolded thermolabile proteins, leaving HSFA1 free to trigger the HSR (Figure 1; Zou et al., 1998; Kim and Schöffl, 2002; Yamada et al., 2007; Hahn et al., 2011). Although thermolabile proteins become denatured and recruit molecular chaperones upon heat exposure, the prevention of Ca^2+^ entry through the plasma membrane led to an absence of HSR (Saidi et al., 2009). In addition, treatment with HSP90 inhibitors triggers a minor HSR at low temperature at rest, whereas a full-blown HSR can be obtained at higher temperatures (Kyle Hadden et al., 2006; Westerheide et al., 2006; Saidi et al., 2009). Therefore, even if all HSP90s are dissociated from HSFA1s, a large fraction of HSFA1s is required to be activated independently from a non-elucidated signal, which strictly depends on the calcium entry across the plasma membrane (Figure 1).

### Histones and DNA Regulation Lead to the Transcription of HSPs

The expression of HSPs in plants is regulated by epigenetics, such as DNA methylation, histone modification, and chromatin remodeling (Boyko et al., 2010; Gao et al., 2014; Gallego-Bartolomé, 2020; Zhao et al., 2021). HSPs gene must be unwrapped from histones to become actively transcribed when HSF1As are bound to HSEs (Figure 1). Following HS, a global rearrangement of the chromatin has been observed in rice, rye, and *A. thaliana* (Santos et al., 2011; Tomás et al., 2013; Probst and Mittelsten Scheid, 2015). These results indicate that, at low temperature, HSPs genes are compacted by histones, and chemical modifications are required for HSPs expression to be allowed under HS. Interestingly, in *A. thaliana*, actin-related protein 6 (ARP6) has been reported to be an essential component of the chromatin remodeling complex required for H2A.Z incorporation (Figure 1; March-Díaz and Reyes, 2009). The *Atarp6* mutant exhibited a higher accumulation of HSP70 at 12, 22, and 27°C (Kumar and Wigge, 2010; Cortijo et al., 2017). Histone chemical modifications, such as methylation and acetylation, have been also indicated to be important for the regulation of HSPs in plants (Lämke et al., 2016; Yamaguchi et al., 2021). Regarding non-coding RNA (ncRNA), such as microRNAs (miRNAs) and small interfering RNAs (siRNAs), its involvement in the regulation of the HSR has been suggested in several land plant species (Khraiwesh et al., 2012; Li et al., 2014; Zhao et al., 2016; Liu et al., 2017; Lin J. S. et al., 2018; Zhao et al., 2021). Thus, following a short period of HS, epigenetic processes have also been implicated in developing a transcriptional heat memory (Bäurle and Trindade, 2020).

## The Role of Small Heat Shock Proteins Under Heat Stress

Among HSP chaperones, the HSP20 (sHSP) family is the most responsive to heat, whereas, at low temperatures, it is mainly repressed (Waters et al., 1996; Sun et al., 2002; Vierling, 2003; Guihur et al., 2020). HSP20s proteins are composed of subunits between 12 and 43 kDa and have an alpha-crystalline domain suggested to bind denatured proteins (Haslbeck et al., 2005; Basha et al., 2012; Waters and Vierling, 2020). *A. thaliana* contains 19 HSP20 (sHSPs) divided into six classes according to their localization (Sun et al., 2002; Waters and Vierling, 2020). Following HS, heat-labile proteins can be bound by HSP20s, in an ATP-independent manner, and folded into their native state by chaperone machinery, thereby preventing further heat-caused denaturation (Waters et al., 1996; Glover and Lindquist, 1998; Veinger et al., 1998; Goloubinoff et al., 1999; Swindell et al., 2007; Haslbeck and Vierling, 2015; Mogk et al., 2015; Mogk and Bukau, 2017). As shown initially in *E. coli*, the small HSP IbpB has been revealed to interact with HSP40, HSP60, and HSP70 chaperone complexes and assist in protein refolding (Veinger et al., 1998). Similar observations have been made in *Pisum sativum* and *Synechocystis sp.* (Mogk et al., 2003). In addition, HSP20s stabilize lipid bilayers and thereby protect the plasma membrane from high fluidity under excessive temperatures (Horváth et al., 2008; Haslbeck and Vierling, 2015).

HSP20s accumulation is essential for basal thermotolerance and the onset of AT in plants. In *A. thaliana*, an *AtHSP17.6II* mutant was unable to establish the AT, whereas the overexpression of LimHSP16.45 from *Lilium davidii* rescued the sensibility to HS (Yang et al., 2020). Supporting these observations, *A. thaliana* RNAi lines of six cytosolic HSP20s showed higher thermosensitivity, whereas the HSP20s overexpression lines restored the phenotype (McLoughlin et al., 2016). In wheat, chloroplastic HSP26 was shown to be required for seed maturation, germination, and development of HS tolerance (Chauhan et al., 2012). Similar observations have been described in other plant species, such as in tobacco and rice (Lee et al., 2000; Zhang et al., 2016). Besides providing protection against noxious temperatures, HSP20s also confer resistance to salt, drought, and cold stresses (Sun et al., 2002; Sarkar et al., 2009; Song and Ahn, 2010; Yang et al., 2014). HSP20s were also described to play key roles in somatic embryogenesis, pollen development, and seed germination (Sun et al., 2002; Volkov et al., 2005; Chauhan et al., 2012).

**FIGURE 2:**
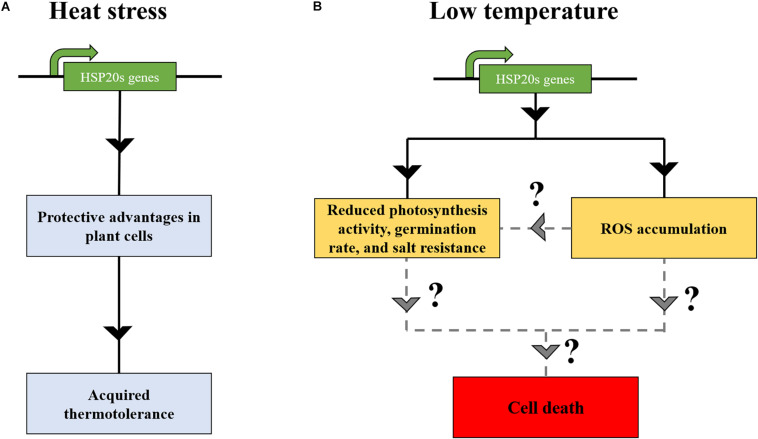
The expression of HSP20s at low temperature might induce deleterious effects in land plants. **(A)** Under heat stress, HSP20s are accumulated, conferring protective advantages in plant cells, leading to the onset of acquired thermotolerance. **(B)** The overexpression of HSP20s at low temperatures might induce deleterious effect on plant growth and development (Sun et al., 2016), such as ROS accumulation (Ma et al., 2019), eventually resulting in the cell death (Sun et al., 2016; Ma et al., 2019). Solid lines indicate the consequences of HSP20s expression at low temperature, whereas dashed lines indicate potential cell death effect on plant cells.

In several plant species, transcriptome and proteome-based analyses have demonstrated a nearly total absence of HSP20s at non-HS temperatures (Hernandez and Vierling, 1993; Simões-Araújo et al., 2003; Finka et al., 2012; Guihur et al., 2020). In contrast, other HSP chaperones families might have a substantial constitutive expression (Finka et al., 2012; Guihur et al., 2020). This raises a question of why plants tightly suppress HSP20s synthesis at non-HS temperature (low temperature) The complete HSP20 repression suggests that its constitutive expression would be problematic (Figure 2). To date, one study has reported a deleterious effect of one HSP20 in *A. thaliana*. *A. stolonifera* HSP17 overexpression in *A. thaliana* led to a reduction in leaf chlorophyll content and photosynthesis activity at both 22 and 40°C. The mutant showed hypersensitive response to exogenous abscisic acid and salinity during germination and during post-germinative growth (Sun et al., 2016). AtHSP24.7 has been described as a central activator of temperature-dependent seed germination (Ma et al., 2019). AtHSP24.7 overexpression accelerated seed germination and caused the accumulation of reactive oxygen species (ROS). In the study of Ma et al. (2019), an absence of negative physiology impact on plants was observed. Yet, it remains to demonstrate that other HSP20 family members behave similarly to HSP24.7, which could increase ROS content and, thereby, inducing apoptosis when achieving a critical threshold. Other related studies have indicated that the overaccumulation of HSP molecular chaperones might be deleterious for plants. For instance, although the overexpression of HSP70-1 improved basal thermotolerance in *A. thaliana*, it resulted in a dwarf phenotype, altering root growth (Sung and Guy, 2015). Furthermore, overexpression lines of HSP90.2, HSP90.5, and HSP90.7 reduced the resistance to salt and drought stress and produced a lower germination rate and lower fresh weight (Song et al., 2009). Thus, plants seem to have established a sophisticated mechanism to tightly regulate the expression of HSP chaperones, presumably to not affect plant fitness (Figure 2).

## Conclusion

In recent years, the threat of global warming and the wide-reaching implications of the adverse effects on plant growth and crop yields have called for more studies about HS. This review has described some aspects of the heat perception and molecules involved in the signaling, ultimately triggering the accumulation of protective HSPs. There is strong evidence in literature showing that the plasma membrane, embedded with CNGCs, acts as a central hub for the perception of incremental temperature. Yet, an unidentified signal, potentially involving calmodulins and kinases, triggers the translocation of HSFA1s into the nucleus to activate essential regulatory responses, such as histone and DNA regulation (Figure 1). To further investigate the heat shock signaling pathway, several questions remain unanswered; among them are the following:

- What are the missing partners involved in the heat signal transduction between CNGCs and the activation of HSFA1 upon HS?
- How are CNGCs subunits assembled to sense and respond to a wide temperature scale?
- Does the overexpression of HSP20s at low temperature induce deleterious phenotypes in land plants?

All these issues need further research to address a comprehensive picture of heat sensing and AT.

## Author Contributions

BB made the figures and AG has updated Figure 1. Both the authors conceived the central ideas of the manuscript, interpreted data from literature, contributed to writing, reviewed, edited, and approved its final version of the manuscript.

## Conflict of Interest

The authors declare that the research was conducted in the absence of any commercial or financial relationships that could be construed as a potential conflict of interest.

## Publisher’s Note

All claims expressed in this article are solely those of the authors and do not necessarily represent those of their affiliated organizations, or those of the publisher, the editors and the reviewers. Any product that may be evaluated in this article, or claim that may be made by its manufacturer, is not guaranteed or endorsed by the publisher.

## Abbreviations

ARP6: actin-related protein 6
AT: acquired thermotolerance
CaM: calmodulin
CaMBD: calmodulin-binding domain
CBK3: calmodulin-binding protein kinase 3
CML: calmodulin-like protein
CNBD: cyclic nucleotide-binding domain
CNGC: cyclic nucleotide-gated channels
ER: endoplasmic reticulum
IQ: isoleucine-glutamine
HS: heat stress
HSE: heat shock element
HSF1: heat shock transcription factor
HSP: heat shock protein
HSR: heat shock response
miRNAs: microRNAs
ncRNA: non-encoding RNA
ROS: reactive oxygen species
siRNAs: small interfering RNAs.
